# Association between *MMP1* -1607 1G>2G Polymorphism and Head and Neck Cancer Risk: A Meta-Analysis

**DOI:** 10.1371/journal.pone.0056294

**Published:** 2013-02-18

**Authors:** Caiyun Zhang, Xicheng Song, Minhui Zhu, Song Shi, Meng Li, Lei Jin, Juntian Lang, Guojun Li, Hongliang Zheng

**Affiliations:** 1 Department of Otorhinolaryngology-Head and Neck Surgery, Changhai Hospital, Second Military Medical University, Shanghai, China; 2 Department of Otolaryngology-Head and Neck Surgery, Yuhuangding Hospital of Qingdao University, Yantai, China; 3 Department of Stomatology, Jinling Hospital, School of Medicine, Southern Medical University, Nanjing, China; 4 Department of Otorhinolaryngology-Head & Neck Surgery, Shanghai Changzheng Hospital, Second Military Medical University, Shanghai, China; 5 Department of Head and Neck Surgery, Unit 1445, The University of Texas MD Anderson Cancer Center, Houston, Texas, United Sates of America; University of California, Irvine, United States of America

## Abstract

**Background:**

MMP1 is an important member of the MMP endopeptidase family that plays a critical role in the development of head and neck cancer (HNC). Several studies have investigated the association between the *MMP1* -1607 1G>2G polymorphism and risk of HNC, but their results have been inconsistent. Here, we conducted a meta-analysis to further explore the role of the *MMP1* -1607 1G>2G polymorphism in HNC development.

**Methods:**

We identified all eligible studies in the electronic databases of PubMed, ISI Web of Knowledge, MEDLINE, Embase, and Google Scholar (from January 2000 to June 2012). A meta-analysis was performed to evaluate the association between the *MMP1* -1607 1G>2G polymorphism and risk of HNC by calculating odds ratios (OR) and 95% confidence interval (CIs).

**Results:**

Twelve studies were included in this meta-analysis. In overall comparison, significant associations were found using the recessive and allelic contrast models (OR, 1.38; 95% CI, 1.07–1.79 and OR, 1.27; 95% CI, 1.05–1.53, respectively), but no association was detected using the dominant model. In the stratified analyses by several variables, significant associations were observed using the recessive, dominant, and allelic contrast models in the Asian population (OR, 1.64; 95% CI, 1.29–2.08; OR, 1.39; 95% CI, 1.06–1.82; and OR, 1.41; 95% CI, 1.21–1.65, respectively), European population (OR, 0.58; 95% CI, 0.40–0.84; OR, 0.64; 95% CI, 0.44–0.92; and OR, 0.68; 95% CI, 0.54–0.85, respectively), and population-based subgroup (OR, 1.24; 95% CI,1.05–1.47; OR,1.48; 95% CI,1.04–2.12; and OR, 1.22; 95% CI, 1.07–1.38, respectively). Furthermore, significant associations were detected in oral cavity cancer and nasopharyngeal cancer under the recessive model.

**Conclusion:**

Our results suggest that the *MMP1* -1607 1G>2G polymorphism is associated with risk of HNC and that it plays different roles in Asian and European populations. Further studies with large sample size are needed to validate our findings.

## Introduction

Head and neck cancer (HNC) globally comprises tumors of the oral cavity, nasopharynx, oropharynx, hypopharynx, and larynx, and is the sixth most common cancer in the world [1]. It is associated with a moderately high recurrence rate, a low survival rate, a high frequency of second primary malignancy (SPM), and a high prevalence of comorbidities [2]. This disease is highly aggressive and can cause significant morbidity [3]. Although tobacco use, alcohol consumption, and viral infection play a major role in the etiology of HNC [4]–[6], only a fraction of these subjects develop HNC, indicating that genetic susceptibility may also contribute to its development [7].

Matrix metalloproteinase-1 (MMP1) might serve as an important molecular marker for HNC. MMP is a family of zinc-dependent endopeptidases that are able to degrade essentially all extracelluar matrix (ECM) components, such as basement membranes, collagen, and fibronectin [8]–[10]. The human MMPs family, which consists of at least 26 proteases, can be divided into several subgroups according to their structure and substrate specificity [11], [12]. These subfamilies include collagenases, gelatinases, stromelysins, matrilysins, and membrane-type MMPs (MT-MMPs), among others. MMPs play an important role in both physiological and pathological conditions, including tissue regeneration, wound repair, reproduction, arthritis, atherosclerosis, and autoimmune blistering disorders of the skin [13]. MMPs have also been implicated in carcinogenesis because of their ability to degrade ECM, which is a key event in cancer progression [14]. Growing evidence has shown that MMPs can facilitate tumor growth, invasion, and metastasis in various cancers [14].

MMP1 (Collagenase-1), located on chromosome 11q22, is an important member of the MMP family that specifically degrades a major component of the ECM, type I collagen, as well as other fibrillar collagens of types II, III, V, and IX [15]–[16]. The *MMP1* gene is expressed in various kinds of normal cells, often at low levels under physiological conditions. However, *MMP1* gene expression increases dramatically in a large number of malignancies, including HNC [17].

The promoter region of *MMP1* plays a critical role in the regulation *MMP1* gene transcription. Within this region, a functional single-nucleotide polymorphism (SNP), *MMP1* -1607 1G>2G (rs1799750), has been identified [18]. It has been reported that *MMP1* -1607 1G>2G contains a guanine insertion/deletion polymorphism at position -1607, which is relative to the transcriptional start site, and could result in higher expression of *MMP1* [19]. Several molecular epidemiological studies have examined the association between the *MMP1* -1607 1G>2G polymorphism and risk of HNC [20]–[30]; however, the results have been inconsistent. To further explore the role of the *MMP1* -1607 1G>2G polymorphism in risk of HNC, we performed a meta-analysis by collecting and analyzing the genotyping data from all eligible case–control studies published to date.

## Materials and Methods

### Search Strategy

To identify all eligible case-control studies that examined the association between *MMP1* polymorphism and risk of HNC (between January 2000 and June 2012), we conducted key word searches in the electronic databases of PubMed, ISI Web of Knowledge, MEDLINE, Embase, and Google Scholar. The key words we used included “head and neck cancer”, “oral cancer”, “pharyngeal cancer”, “hypopharyngeal cancer”, or “laryngeal cancer”, and “MMP1”, “Matrix Metalloproteinase1”, “collagenase”, and “polymorphism”, “variant”, “genotype”, or “SNP”. We also performed a manual search of the references of all identified articles in order to find additional studies. If important data were not reported in the original articles, we would contact with authors directly. Abstracts, unpublished reports and articles written in non-English languages were excluded.

### Data Extraction

All data extraction was performed by two independent investigators, and they reached a consensus on all items by discussion. The following information was extracted from each included study: first author, published year, ethnicity of study population (Asian or European), numbers of case and controls, genotype distribution, genotyping methods, allele, etc.

### Inclusion and Exclusion Criteria

Eligible studies met the following criteria: (1) studies on the association between the *MMP1*-1607 1G>2G polymorphism and risk of head and neck cancer, (2) case-control studies, (3) studies with sufficient available data to calculate an odds ratio (OR) with a 95% confidence interval (CI) and P -value, and (4) studies published in English. Studies with insufficient information about genotype frequency or number were not included. If the same population was included, with overlapping data, in more than one study, only the most recent or complete study was included in the meta-analysis.

### Statistical Analysis

First, we tested Hardy-Weinberg equilibrium (HWE) by comparing the expected and observed genotype frequencies of the control group using the Pearson chi-square test for goodness of fit. The association between the *MMP1*-1607 1G>2G polymorphism and risk of HNC was assessed by OR and 95% CI. Heterogeneity between studies was evaluated by Cochran’s χ2 -based Q statistic test. If the P -value for heterogeneity was <0.05, suggesting that there was obvious heterogeneity of the data, a random-effects model was used; otherwise, we used a fixed-effects model to pool the results. The I^2^ test was also used to estimate the extent of heterogeneity between studies. As a guide, I^2^ values of <25% were considered “low”, value of ∼50% were considered “moderate”, and values of >75% were considered “high” [31]. We explored the association between the *MMP1*-1607 1G>2G polymorphism and risk of HNC using a recessive genetic model (2G/2G versus 1G/1G+1G/2G), a dominant genetic model (2G/2G+1G/2G versus 1G/1G), and an allelic contrast model (2G allele versus 1G allele). In addition to an overall comparison, we also performed subgroup analyses based on the ethnicity of the study population, the source of the controls, and tumor site. The significance of pooled ORs was detected by the Z test (P<0.05 was considered significant). We investigated publication bias using the funnel plot and Egger’s test. Sensitivity analyses were also applied to assess the stability of the results by repeating the meta-analysis, omitting each study one at a time. All P values were two-sided, and all statistical analyses were carried out using STATA 11.0 software (Stata Corporation, College Station, TX).

## Results

### Study Characteristics

**Figure 1:**
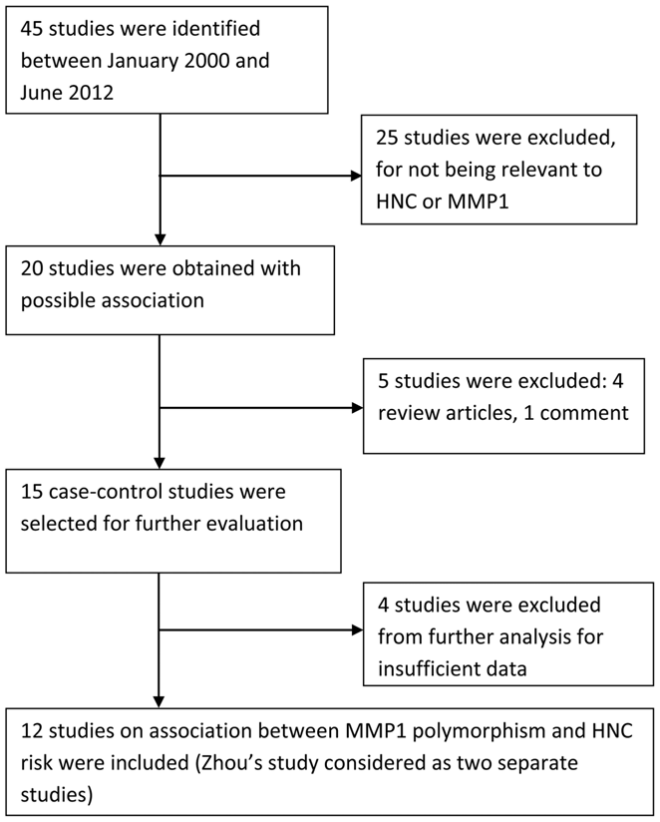
Flow diagram of study identification.

Using the search strategy described, we found 45 relevant articles. Thirty-four studies were excluded for not meeting the inclusion/exclusion criteria: 25 studies were not relevant to HNC or *MMP1*; 4 studies did not have sufficient data for further analysis; 4 studies were review articles; and 1 study was an article of comment. In a case-control study investigating the *MMP1*-1607 1G>2G polymorphism in two independent populations [29], each population was considered as a separate study. Thus, we included 12 studies [20]–[30] in this meta-analysis of the association between *MMP1*-1607 1G>2G polymorphism and risk of HNC (Figure 1). Characteristics of the studies, including publication year, population ethnicity, tumor site, genotype data, and sample size (case/control) are summarized in Table 1. The most commonly used genotyping method in these studies was polymerase chain reaction–restriction fragment length polymorphism (PCR-RFLP). All but one study [25] indicated that genotypic distribution of the controls was consistent with HWE at a statistical significance level of 0.05.

**Table 1:** Characteristics of 12 case-control studies included in this meta-analysis.

First author	Year	Ethnicity	Tumorsite	Sourceofcontrols	Cases				Controls				Genotypingmethod	P_HWE_ 3
					N	2G/2G	1G/2G	1G/1G	N	2G/2G	1G/2G	1G/1G		
Cao	2006	Asian	Oral cavity	HB^1^	96	55	33	8	120	41	54	25	PCR-RFLP	0.359
Vairaktaris	2007	European	Oral cavity	HB	156	52	68	36	141	60	57	24	PCR-RFLP	0.109
Hashimoto	2004	Asian	Mixed HNC	HB	140	75	48	17	223	95	104	24	PCR-RFLP	0.571
Nishizawa	2007	Asian	Oral cavity	PB^2^	170	77	79	14	164	64	71	29	PCR-RFLP	0.235
O-charoenrat	2006	Asian	Mixed HNC	HB	300	149	97	54	300	89	150	61	PCR-RFLP	0.879
Shimizu	2008	Asian	Oral cavity	HB	69	37	22	10	91	36	46	9	PCR-RFLP	0.3
Zinzindohoue	2004	European	Mixed HNC	HB	125	18	66	41	249	66	126	57	PCR-RFLP	0.833
Lin	2004	Asian	Oral cavity	HB	121	57	54	10	147	63	60	24	PCR	0.14
Kondo	2005	Asian	Nasopharynx	HB	83	41	32	10	82	19	44	19	PCR-RFLP	0.508
Nasr	2007	African	Nasopharynx	PB	174	98	63	13	171	83	63	25	PCR-RFLP	0.029
Zhou	2007	Asian	Nasopharynx	PB	591	241	285	65	479	183	235	61	PCR	0.281
Zhou	2007	Asian	Nasopharynx	PB	238	113	96	29	280	110	132	38	PCR	0.872

1 HB: hospital-based,

2 PB: population-based,

3 HWE: Hardy–Weinberg equilibrium.

### Quantitative Data Synthesis

**Figure 2:**
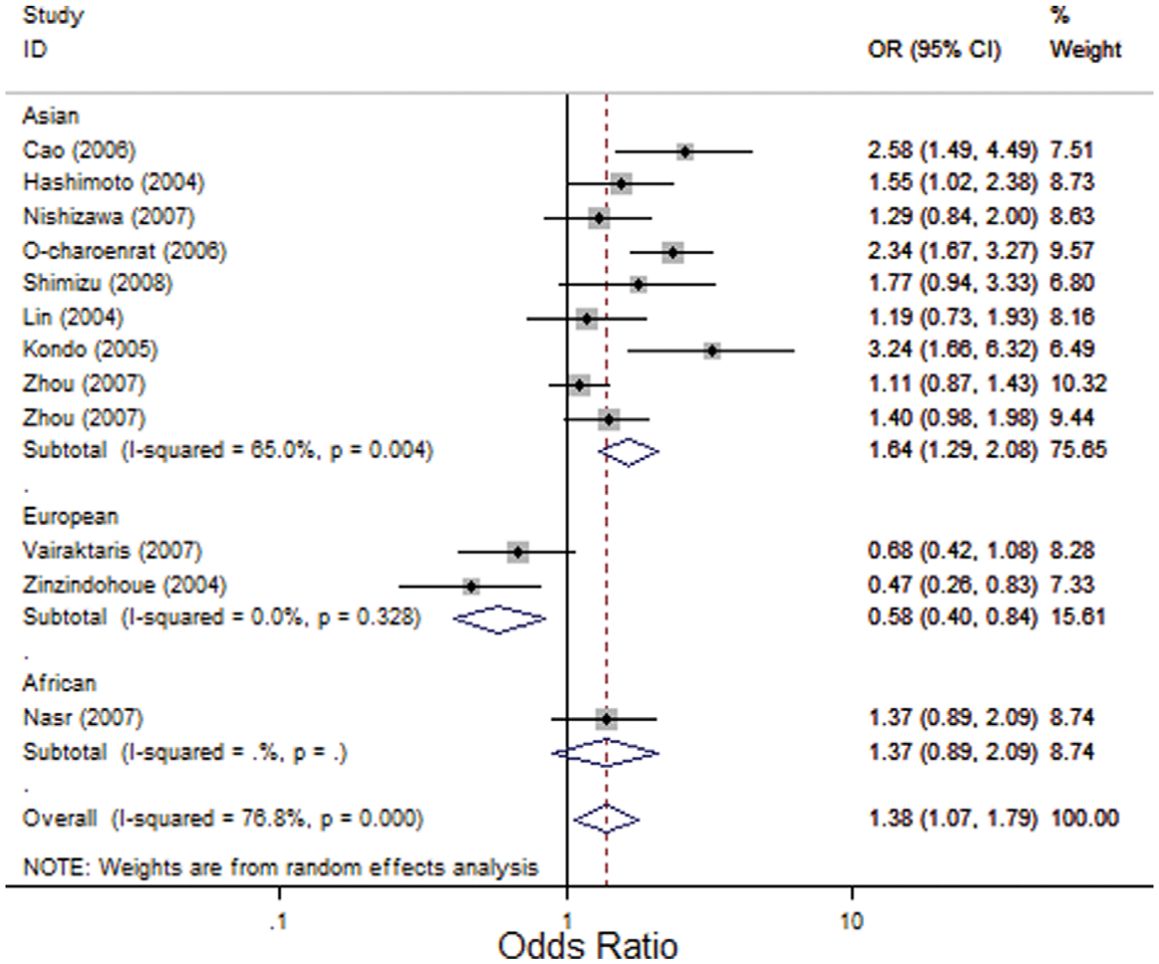
Forest plot for association between *MMP1*-1607 1G>2G and risk of HNC under the recessive model (2G/2G VS 1G/1G+1G/2G). A random effects model was used. The *squares* and *horizontal lines* represent the study-specific OR and 95% CI. The *diamonds* correspond to the summary OR and 95% CI.

**Figure 3:**
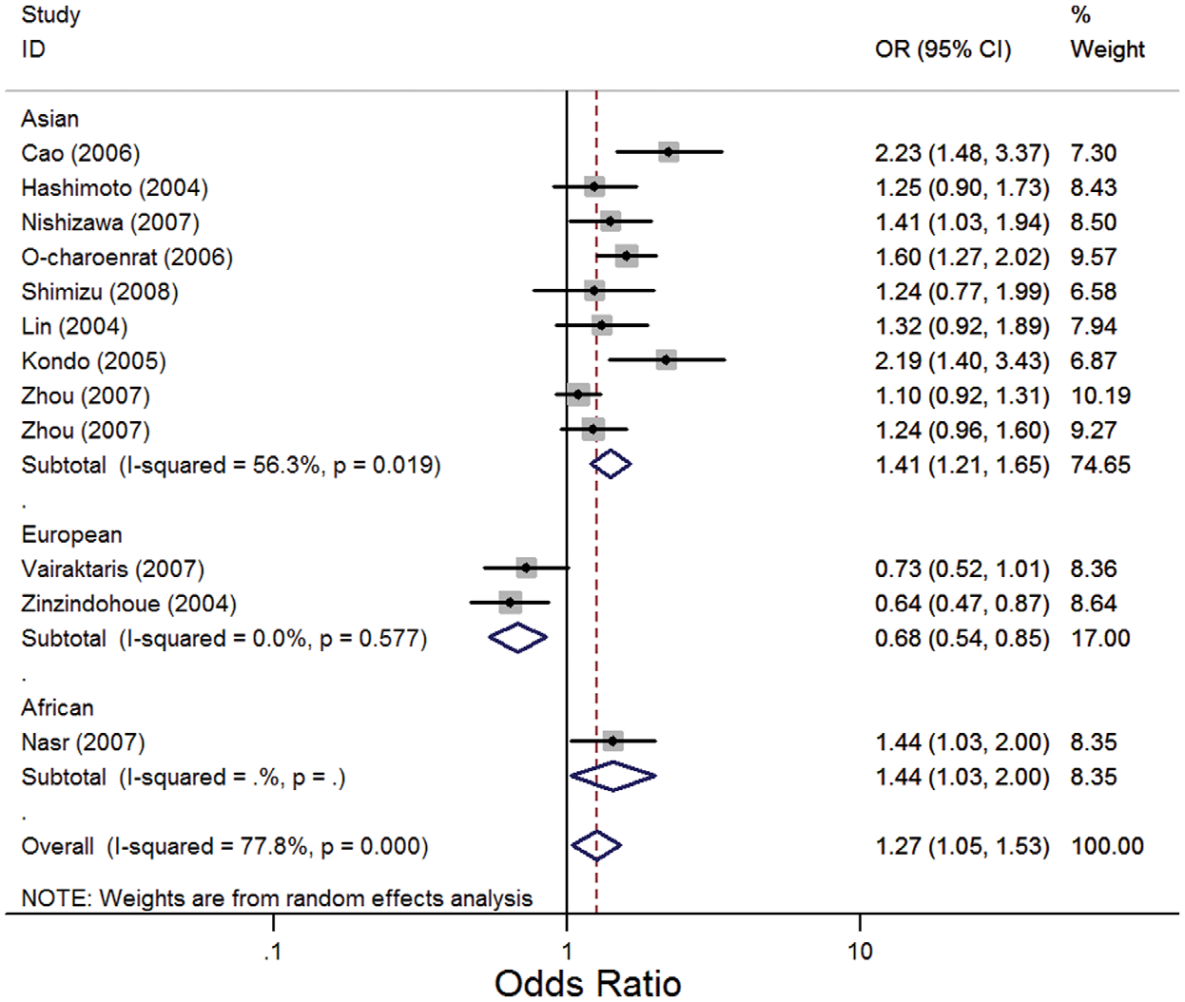
Forest plot for association between *MMP1*-1607 1G>2G and risk of HNC under the allelic contrast model (2G VS 1G). A random effects model was used. The *squares* and *horizontal lines* represent the study-specific OR and 95% CI. The *diamonds* correspond to the summary OR and 95% CI.

As shown in Table 2, significant associations between the *MMP1* -1607 1G>2G polymorphism and risk of HNC were found in overall comparisons using the recessive model (OR, 1.38; 95% CI, 1.07–1.79; I^2^, 76.8%, *P_heterogeneity_*<0.001) (Figure 2) and the allelic contrast model (OR, 1.27; 95% CI, 1.05–1.53; I^2^, 77.8%, *P_heterogeneity_*<0.001) (Figure 3), but no significant association was observed using the dominant model (OR, 1.25; 95% CI, 0.94–1.66; I^2^, 61.8%, *P_heterogeneity_ = *0.002). Similarly, in an analysis of HWE studies, significant associations were found using the recessive model (OR, 1.39; 95% CI, 1.04–1.84; I^2^, 78.9%, *P_heterogeneity_*<0.001) and the allelic contrast model (OR, 1.25; 95% CI, 1.02–1.54; I^2^, 79.4%, *P_heterogeneity_*<0.001), and no significant association was observed using the dominant model (OR, 1.20; 95% CI, 0.90–1.60; I^2^, 61.5%, *P_heterogeneity_* = 0.004).

**Table 2:** Stratified analysis of *MMP1* -1607 1G>2G polymorphism and HNC risk.

Variables	N^a^	Recessive model (2G2G VS 1G2G+1G1G)			Dominant model(2G2G+1G2G VS 1G1G)			Allelic contrast model(2G VS 1G)		
		OR (95% CI)	*P* b	I^2^	OR (95% CI)	*P* b	I^2^	OR (95% CI)	*P* b	I^2^
Total	12	1.38^c^(1.07, 1.79)*	<0.001	76.8	1.25^c^(0.94, 1.66)	0.002	61.8	1.27^c^(1.05, 1.53)*	<0.001	77.8
HWE	11	1.39^c^(1.04, 1.84)*	<0.001	78.9	1.20^c^(0.90, 1.60)	0.004	61.5	1.25^c^(1.02, 1.54)*	<0.001	79.4
Ethnicity										
Asian	9	1.64^c^(1.29, 2.08)*	0.004	65	1.39(1.06, 1.82)*	0.084	42.6	1.41^c^(1.21, 1.65)*	0.019	56.3
European	2	0.58(0.40, 0.84)*	0.328	0	0.64(0.44, 0.92)*	0.759	0	0.68(0.54, 0.85)*	0.577	0
African	1	1.37(0.90, 2.09)	N/A	N/A	2.12(1.05, 4.30)	N/A	N/A	1.44(1.03, 2.00)	N/A	N/A
Source of control										
HB^d^	8	1.45^c^(0.93, 2.24)	<0.001	84	1.12^c^(0.76, 1.66)	0.005	65.9	1.27^c^(0.93, 1.74)	<0.001	84.9
PB^e^	4	1.24(1.05, 1.47)*	0.702	0	1.48(1.04, 2.12)*	0.159	42.1	1.22(1.07, 1.38)*	0.375	3.6
Tumor site										
Oral cavity	8	1.43^c^(1.00, 2.04)*	0.001	70.9	N/A	N/A	N/A	N/A	N/A	N/A
Oropharynx/hypopharynx	3	1.26^c^(0.42, 3.79)	0.001	85.8	N/A	N/A	N/A	N/A	N/A	N/A
Larynx	3	1.28^c^(0.53, 3.10)	0.018	75.2	N/A	N/A	N/A	N/A	N/A	N/A
Nasopharynx	4	1.47^c^(1.05, 2.05)*	0.031	66.1	N/A	N/A	N/A	N/A	N/A	N/A

a Number of comparisons,

b P-value for Q-test.

c Random-effects model was used when P-value of Q-test for heterogeneity <0.05; otherwise fixed-effects model was used.

d HB: hospital-based,

e PB: population-based,

* Statistically significant, with P<0.05.

Interestingly, in stratified analysis by ethnicity, we found that this polymorphism played different roles in Asian and European populations. In the European population, the *MMP1* -1607 1G>2G polymorphism had significant protective effects on the risk of HNC in all three genetic models (OR, 0.58; 95% CI, 0.40–0.84; I^2^ = 0, *P_heterogeneity_* = 0.328 for the recessive model; OR, 0.64; 95% CI, 0.44–0.92; I^2^ = 0, *P_heterogeneity_* = 0.759 for the dominant model; and OR, 0.68; 95% CI, 0.54–0.85; I^2^ = 0, *P_heterogeneity_* = 0.577 for the allelic contrast model), whereas in Asian population, it increased the risk of HNC significantly in all three models (OR, 1.64; 95% CI, 1.29–2.08; I^2^, 65%, *P_heterogeneity_* = 0.004 for the recessive model; OR, 1.39; 95% CI, 1.06–1.82; I^2^, 42.6%, *P_heterogeneity_* = 0.084 for the dominant model; and OR, 1.41; 95% CI, 1.21–1.65; I^2^, 56.3%, *P_heterogeneity_*<0.019 for the allelic contrast model) (Figure 2, 3).

Stratification based on the source of the controls showed significant associations between the *MMP1* -1607 1G>2G polymorphism and risk of HNC in the population-based subgroup (OR, 1.24; 95% CI, 1.05–1.47; I^2^, 0, *P_heterogeneity_* = 0.702 for the recessive model; OR, 1.48; 95% CI, 1.04–2.12; I^2^, 42.1%, *P_heterogeneity_* = 0.159 for the dominant model; and OR, 1.22; 95% CI, 1.07–1.38; I^2^, 3.6%, *P_heterogeneity_* = 0.375 for the allelic contrast model). However, no significant association was found in the hospital-based subgroup (OR, 1.45; 95% CI, 0.93–2.24; I^2^, 84%, *P_heterogeneity_*<0.001 for the recessive model; OR, 1.12; 95% CI, 0.76–1.66; I^2^, 65.9%, *P_heterogeneity_* = 0.005 for the dominant model; and OR, 1.27; 95% CI, 0.93–1.74; I^2^, 84.9%, *P_heterogeneity_*<0.001 for the allelic contrast model).

In the stratified analysis based on tumor site, significant associations were found in the recessive model for oral cavity cancer (OR, 1.43; 95% CI, 1.00–2.04; I^2^, 70.9%, *P_heterogeneity_* = 0.001) and nasopharyngeal cancer (OR, 1.47; 95% CI, 1.05–2.05; I^2^, 66.1%, *P_heterogeneity_* = 0.031).

However, no significant association was found for either pharyngeal (oropharynx/hypopharynx) cancer (OR, 1.26; 95% CI, 0.42–3.79; I^2^, 85.8%, *P_heterogeneity_* = 0.001) or laryngeal cancer (OR, 1.28; 95% CI, 0.53–3.10; I^2^, 75.2%, *P_heterogeneity_* = 0.018).

### Heterogeneity Analysis

In this study, significant heterogeneity was found in all three genetic models. However, when the population were stratified by ethnicity, heterogeneity disappeared in the European population (I^2^ = 0, *P_heterogeneity_* = 0.328 for the recessive model; I^2^ = 0, *P_heterogeneity_* = 0.759 for the dominant model; and I^2^ = 0, *P_heterogeneity_* = 0.577 for the allelic contrast model) and decreased significantly in the Asian population under the dominant model (I^2^ = 42.6%, *P_heterogeneity_* = 0.084). Similarly, stratification based on the source of the controls significantly reduced the heterogeneity in the population-based subgroups (I^2^ = 0, *P_heterogeneity_* = 0.702 for the recessive model; I^2^ = 42.1%, *P_heterogeneity_* = 0.159 for the dominant model; and I^2^ = 3.6%, *P_heterogeneity_* = 0.375 for the allelic contrast model).

### Sensitivity Analysis

**Figure 4:**
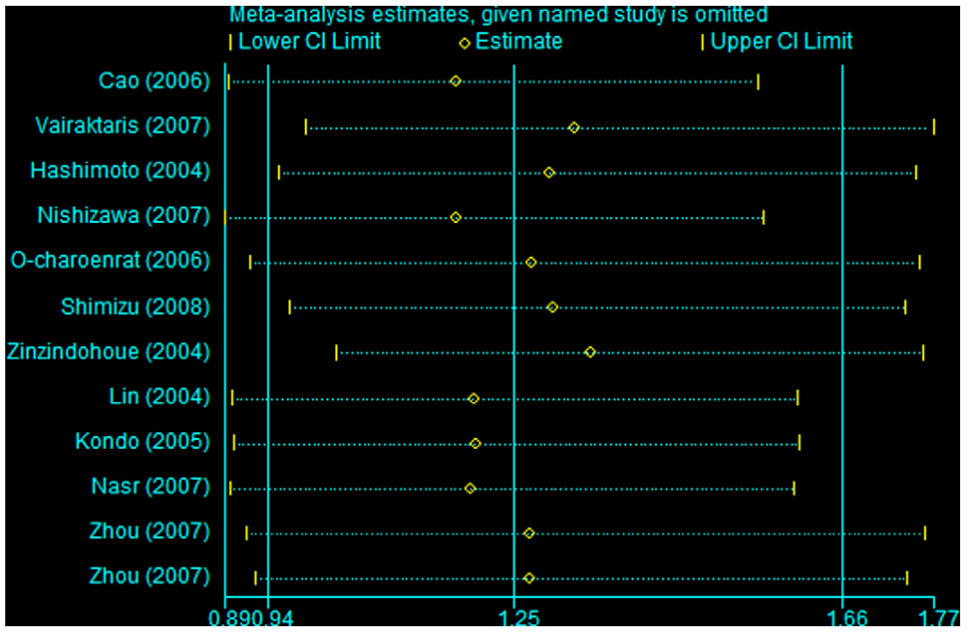
Sensitivity analysis through deletion of one study at a time to reflect the influence of the individual dataset to the pooled ORs under the dominant model.

Sensitivity analyses were performed to determine the influence of the individual dataset on the pooled ORs by sequential removal of each eligible study. The results indicated a borderline increase in risk after excluding Zinzindohoue’s study [30] in a dominant model (Figure 4). In contrast, in the recessive genetic and allelic contrast models, the significance of the pooled ORs was not influenced by any single study (data not shown), suggesting that our results are statistically robust.

### Publication Bias

**Figure 5:**
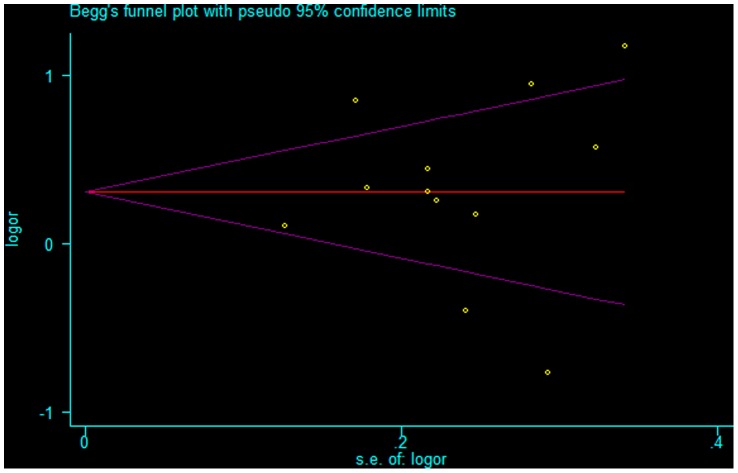
Begg’s funnel plot for publication bias test. Each point represents a separate study for the indicated association under the recessive model.

Begg’s funnel plot and Egger’s test were performed to assess the publication bias of the literature. The shapes of the funnel plots in all the genetic models did not reveal any evidence of obvious asymmetry (see Figure 5 for a representative funnel plot of the recessive model). Furthermore, Egger’s test did not show any statistical evidence of publication bias (P = 0.757 for the recessive model, P = 0.204 for the dominant model, and P = 0.442 for the allelic contrast model).

## Discussion

In this first meta-analysis of the association between the *MMP1* -1607 1G>2G polymorphism and risk of HNC, we found significant associations in the overall comparison using the recessive and allelic contrast models. Individuals with the 2G/2G genotype or 2G allele carriers could have an increased risk of HNC. Moreover, in the stratified analyses by several variables, including ethnicity, source of the controls, and tumor site, significant associations were observed in the Asian population, European population, population-based control subgroups, oral cavity cancer, and nasopharyngeal cancer. Although our analysis had relatively small sample size, with current sample size, we, however, had power to detect a reasonable degree of association. These results suggest that the *MMP1* -1607 1G>2G polymorphism might modulate genetic susceptibility to HNC.

MMP1, a major member of the MMPs family, has been implicated in the development of a variety of cancers because of its ability to degrade ECM [14]. The expression level of the *MMP1* gene can increase in various tumors, which has been associated with a poor prognosis in some types of cancers [32]–[34]. Moreover, the promoter region of the *MMP1* gene can influence its expression. Rutter et al. first described the polymorphism at -1607 in the *MMP1* promoter [18]. It has been demonstrated that the *MMP1* -1607 1G>2G polymorphism is associated with increased transcription of the *MMP1* gene which is attributed to its 2G allele creating a core-binding site for the Ets transcription factor family, resulting in a higher expression level of *MMP1* [19].

In this meta-analysis, we found that individuals with the 2G/2G genotype had a higher risk of developing HNC under a recessive model, but no association was observed under a dominant model, which implies that homozygous 2G may have a stronger effect on an individual’s phenotype than heterozygous 2G, and thus, 2G/2G genotype carriers may be more susceptible to the development of HNC than 1G/2G or 1G/1G genotype carriers. Similarly, we also found that under the allelic contrast model 2G allele carriers had a higher risk of HNC than 1G allele carriers. This finding suggests that the 2G allele may increase susceptibility to HNC because of its association with increased transcription of the *MMP1* gene. However, this hypothesis needs to be tested in future studies.

A few of the source studies also reported results linking the 2G allele to an increased risk of HNC. O-charoenrat et al [27] found that cell lines with 2G/2G genotype expressed a higher level of *MMP1* mRNA than other genotypes and individuals with the 2G/2G genotype had a higher risk of HNC, suggesting that the *MMP1* 2G allele may be a risk factor that could increase susceptibility to HNC. Cao et al. investigated the role of the *MMP1* -1607 1G>2G polymorphism in oral squamous cell carcinoma (OSCC) and reported that the 2G allele increased significantly in OSCC patients when compared to controls, indicating that the *MMP1* -1607 1G>2G polymorphism may be associated with risk of OSCC in a Chinese population [20]. Similarly, Nishizawa et al. explored the association between *MMP1* -1607 1G>2G and risk of OSCC in a Japanese population and found that the frequency of 2G alleles was significantly higher than that of 1G allele in OSCC patients [26]. They concluded that the *MMP1* 2G allele might play a crucial role in the early onset of OSCC. However, Zhou et al. reported that no significant association between the *MMP1* -1607 1G>2G polymorphism and risk of HNC was found in two different populations [29]. These inconsistent results may be attributed to differences in genetic backgrounds, environmental factors, and other factors, such as small sample size or inadequate adjustment for confounding factors.

Interestingly, our subgroup analysis by ethnicity showed that the *MMP1* -1607 1G>2G polymorphism played different roles in Asian and European populations. In the European population, it was significantly associated with reduced risk in all three genetic models in European population, whereas in Asian population, it was significantly associated with increased risk. For example, Zinzindohoue et al. examined the association of the *MMP1* -1607 1G>2G polymorphism with risk of HNC in a case-control study in a European population [30]. They found that 2G allele frequency was significantly lower in cases than in controls, and individuals with the homozygous 2G/2G genotype were at lower risk of HNC than those with the 1G/1G genotype. Similarly, Vairaktaris et al. found that the *MMP1* -1607 1G>2G polymorphism was associated with a decreased risk of oral cancer in 2G allele carriers in a European population [21]. In contrast, in Asian populations, most studies found that the *MMP1* -1607 1G>2G polymorphism was associated with an increased risk of HNC in patients with the 2G/2G genotype or 2G allele carriers [20], [22], [23], [27]. These conflicting results may be due to the different genetic backgrounds in these populations, subsequently leading to different genetic susceptibility to the same disease. Moreover, HNC is a disease caused by multiple genetic and environmental factors, and possibly gene–gene and gene–environment interactions. Additionally, other factors such as linkage disequilibrium (LD) may also contribute to this discrepancy [35]. However, because of the limited number of studies in European population and relatively small sample sizes, these results should be interpreted with caution. Further study with larger sample sizes is warranted in different populations.

Heterogeneity is a major problem when interpreting the results of meta-analyses. In this study, significant heterogeneity was detected in overall comparisons using all three genetic models. Ethnicity was an important reason for this heterogeneity. Individuals from different ethnicities may have diverse genetic backgrounds and environmental factors, and consequently, the same polymorphism may play different roles in different populations. Therefore, when we performed stratified analysis by ethnicity, the heterogeneity disappeared in the European population and decreased significantly in the Asian population. Furthermore, the source of the controls was another factor that contributed to heterogeneity. The *MMP1* genotype distributions in population-based controls may be similar to normal, and thus, population-based controls could be more reliable than hospital-based controls. This might partially explain why the results from the stratified analysis by the source of the controls were different between the two subgroups. In addition, another reason for the heterogeneity between studies was the tumor site. In the stratified analysis by tumor site, significant associations were found for oral cavity cancer and nasopharyngeal cancer, but not for either pharyngeal (oropharynx/hypopharynx) cancer or laryngeal cancer. Although HNC includes tumors from different sites, risk factors for these cancers are different. For example, oral cavity and laryngeal cancers are majorly associated with tobacco use and alcohol consumption, while oropharyngeal and nasopharyngeal cancers are principally related to viral infection, such as human papillomavirus (HPV) and Epstein-Barr virus (EBV). Thus, further studies with larger sample size and different tumor sites are warranted.

The present study has some limitations. First, the study number was limited and the total sample size was relatively small; thus, our estimates of association might have occurred by chance. Second, significant heterogeneity was detected in our study, and thus, the results must be interpreted with caution. However, heterogeneity disappeared in some subgroups when stratified analysis was performed. Therefore, the results from the subgroup analyses may be more meaningful, as the polymorphism may play different roles in diverse subgroups. Third, further subgroup stratification based on other risk factors such as alcohol consumption, tobacco smoking and HPV status could not be performed because of the limited data [36]. Fourth, our meta-analysis was based on unadjusted estimates because only 3 original studies provided adjusted estimates, and the adjusted covariates varied among these studies. A more comprehensive analysis should be conducted if detailed information such as environmental factors and lifestyles are available. Finally, we could not conduct a meta-analysis using linkage disequilibrium, as few studies performed haplotypic analysis.

In conclusion, our meta-analysis suggests that the *MMP1* -1607 1G>2G polymorphism is associated with HNC risk. Moreover, subgroup analysis based on ethnicity indicates that it may play different roles in Asian and European populations. However, due to the limited study numbers and relatively small sample sizes, our results should be validated in future studies with larger sample sizes and in different ethnic populations.

## Supporting Information

Checklist S1 **PRISMA 2009 Checklist.**
(DOC)
